# Draft Genome Sequence of Enterococcus faecalis AS003, a Strain Possessing All Three Type II-a CRISPR Loci

**DOI:** 10.1128/MRA.01449-20

**Published:** 2021-03-18

**Authors:** Kevin B. Mulkerrins, Casandra Lyons, Michael P. Shiaris

**Affiliations:** a Shiaris Laboratory, Department of Biology, University of Massachusetts Boston, Boston, Massachusetts, USA; University of Delaware

## Abstract

Enterococcus faecalis is a clinically significant member of the human microbiome. Three CRISPR-Cas loci are located in conserved locations. Previous studies provide evidence that E. faecalis strains with functional CRISPR-Cas genes are negatively correlated with antibiotic resistance. Here, we report the genome sequence of an unusual strain possessing all three CRISPR-Cas loci.

## ANNOUNCEMENT

Enterococcus faecalis is a widespread bacterial species found in environments ranging from soil to animal gut microbiomes (1, 2). An opportunistic pathogen, it is a common etiological agent of nosocomial infection (1, 3). It frequently undergoes horizontal gene transfer (HGT) associated with increased pathogenicity and acquired antibiotic resistance genes (ARGs) (4). Previous investigations of E. faecalis CRISPR loci have demonstrated a negative correlation between functional CRISPR loci and ARGs, presumably because functional CRISPR degrades foreign DNA as an immune response, thus impeding HGT (2, 5).

E. faecalis has three type II-A CRISPR-Cas loci in respectively conserved locations. From most to least common are CRISPR2, CRISPR1-Cas, and CRISPR3-Cas. CRISPR2 lacks *cas* genes and is assumed nonfunctional (6). The CRISPR type is determined by *cas* genes; however, CRISPR2 arrays show sequence similarity to CRISPR1 arrays (2, 6, 7). While enterococcal CRISPR loci can coincide, we have not observed other strains to possess all three; thus, AS003 was selected for sequencing.

Strain AS003 was cultured from an activated sludge sample at the Deer Island wastewater treatment plant in Boston, MA (8). Diluted activated sludge was filtered through 0.22-μm membrane filters and then incubated at 35°C on m-*Enterococcus* agar (Difco). Colonies were streaked for isolation onto Enterococcosel agar (BBL) and grown at 35°C. Extraction cultures were grown in Trypticase soy broth without shaking. DNA extractions were performed using the MoBio UltraClean microbial DNA isolation kit. The variable region of the 16S rRNA gene was amplified using universal bacterial DNA primers (forward, 5′-CCTACGGGAGGCAGCAG-3′; reverse, 5′-ATTACCGCGGCTGCTGG-3′) (9). Isolates were identified by 16S rRNA sequence matches in the Ribosomal Database Project (10). Enterococcus faecalis AS003 sequencing libraries were prepared from genomic DNA using the Nextera XT DNA library preparation kit and then indexed using the Nextera XT V2 index kit (Illumina, San Diego, CA). Libraries were purified and 350- to 500-bp fragments selected using the QIAquick gel extraction kit (Qiagen, Valencia, CA). Sequencing was performed on an Illumina MiSeq platform using a 500-cycle MiSeq V2 reagent kit.

A total of 1,670,410 250-bp reads were obtained for AS003. Genome assembly was performed using the A5-miseq pipeline, which trims and denoises the raw reads and then corrects the misassembled contigs to produce the final scaffolds (11). The assembly *N*_50_ score is 26,565 bp. The scaffolds were annotated by the National Microbial Pathogen Database Resource’s Rapid Annotation using Subsystem Technology (RAST) service (12).

Assembly produced a 2,976,733-bp draft genome sequence consisting of 195 contigs. CRISPR1-Cas occurs on scaffold 115. CRISPR2 occurs on scaffold 25 and is confirmed by the cooccurrence of its flanking genes, cytochrome *d* ubiquinol oxidase subunit I (*cydA*), and transcriptional regulator, the AraC family. CRISPR3-Cas occurs on two scaffolds. The *cas* operon occurs on scaffold 124, and the array, confirmed by flanking gene *pstS*, occurs on scaffold 155. No mobile ARGs were detected in the genome sequence.

The presented draft assembly provides a rare example of an E. faecalis strain possessing all three canonical CRISPR loci. We had not observed the coincidence of CRISPR1 and CRISPR3 in E. faecalis prior to the isolation of this strain. The uncommon occurrence of CRISPR3 is expected to make this combination unlikely; however, a survey into the genome sequences available in public databases may reveal additional strains with both CRISPR1 and CRISPR3 or all three loci.

### Data availability.

This whole-genome shotgun project has been deposited at DDBJ/ENA/GenBank under the accession number JACJUJ000000000. The version described in this paper is the first version, JACJUJ010000000. The BioProject and BioSample accession numbers are PRJNA648426 and SAMN15641373, respectively (13).
